# Combination therapy with copanlisib and ABL tyrosine kinase inhibitors against Philadelphia chromosome-positive resistant cells

**DOI:** 10.18632/oncotarget.10605

**Published:** 2016-07-14

**Authors:** Seiichi Okabe, Tetsuzo Tauchi, Yuko Tanaka, Juri Sakuta, Kazuma Ohyashiki

**Affiliations:** ^1^ Department of Hematology, Tokyo Medical University, Tokyo, Japan

**Keywords:** chronic myeloid leukemia, phosphoinositide 3-kinase, ABL tyrosine kinase inhibitor, feeder cell

## Abstract

ABL tyrosine kinase inhibitor (TKI) therapy has improved the survival of patients with Philadelphia (Ph) chromosome-positive leukemia. However, ABL TKIs cannot eradicate leukemia stem cells. Therefore, new therapeutic approaches for Ph-positive leukemia are needed. Aberrant activation of phosphoinositide 3-kinase (PI3K) signaling is important for the initiation and maintenance of human cancers. Copanlisib (BAY80-6946) is a potent inhibitor of PI3Kα and PI3K-δ. Here we investigated the efficacy of combination therapy of copanlisib with an ABL TKI (imatinib, nilotinib, or ponatinib) using BCR-ABL-positive cells. Although the effects of the ABL TKI treatment were reduced in the presence of the feeder cell line, HS-5, copanlisib inhibited cell growth. Upon combining ABL TKI and copanlisib, cell growth was reduced. Ponatinib and copanlisib combined therapy reduced tumor volume and increased survival in mouse allograft models, respectively. These results indicate that the PI3Kα and -δ inhibitors overcame the chemoprotective effects of the feeder cells and enhanced ABL TKI cytotoxicity. Thus, co-treatment with ABL TKI and copanlisib may be a powerful strategy against ABL TKI-resistant cells, including those harboring the related T315I mutation.

## INTRODUCTION

Chronic myeloid leukemia (CML) is a myeloproliferative neoplasm associated with the presence of the BCR-ABL fusion gene encoded by the Philadelphia (Ph) chromosome [1]. The fusion protein BCR-ABL is the driving force of CML leukemogenesis. BCR-ABL promotes the activation of downstream signaling molecules, increasing cell survival and driving proliferation [2]. Tyrosine kinase inhibitors (TKIs) have improved treatment outcomes for patients with CML. Imatinib, which was the first ABL TKI introduced, induces highly hematologic and cytogenetic responses in patients newly diagnosed with CML in the chronic phase (CP) [3, 4]. Recently, two second-generation ABL TKIs (nilotinib and dasatinib), which are effective in patients resistant to imatinib and intolerant to imatinib, were developed [5, 6]. In two separate phase 3 studies, compared with imatinib, nilotinib and dasatinib were found to have superior efficacy in newly diagnosed patients [7, 8]. Thus, nilotinib and dasatinib were approved for first-line use for CML-CP. However, nilotinib and dasatinib are rarely used in first-line therapy, and imatinib remains the most common first-line therapy for CML-CP. Additionally, imatinib has many fewer side-effects. Although ABL TKI therapy is effective for treating patients with Ph-positive leukemia, some do not achieve an optimal response or they eventually develop TKI resistance. The T315I point mutation in the BCR-ABL kinase domain is recognized as a common mechanism for conferring drug resistance against all available ABL TKIs [9]. Recently, a third-generation ABL TKI ponatinib was developed. Ponatinib is a pan-ABL TKI for patients with Ph-positive leukemia. In a clinical trial, ponatinib has been demonstrated to have anti-leukemic activities among patients with CML and Ph-positive acute lymphoblastic leukemia, including the T315I mutation [10]. However, resistance to ponatinib has also been reported [11]. Furthermore, leukemia stem cells (LSCs) in CML constitute a subpopulation of malignant cells capable of self-renewal and differentiation. LSCs in CML do not depend on BCR-ABL kinase activity for survival [12]. Therefore, late treatment failure may relate to the persistence of CML stem cells. Consequently, alternative strategies are required to improve outcomes for patients with CML.

The phosphoinositide 3-kinase (PI3K) pathway plays an important role in cellular metabolism, growth, survival, and angiogenesis. PI3K exists as a heterodimer formed by catalytic and regulatory subunits. The p110 catalytic subunit of PI3K is expressed as four different isoforms (α, β, γ, and δ). Both p110α and p110β are expressed ubiquitously in tissues, whereas p110δ is primarily present in hematopoietic cells. p110γ is expressed in the pancreas and skeletal muscles [13]. Activation of the PI3K pathway is a occurs frequently in human cancers [14]. In addition, dysregulation of the PI3K signaling pathway has been implicated in conferring resistance to conventional therapy [14]. Additionally, PI3K/Akt/mammalian target of rapamycin (mTOR) signaling is a well-known oncogenic signaling pathway in both BCR-ABL-dependent and BCR-ABL-independent CML. BCR-ABL-independent activation of the PI3K signaling pathway has also been reported in ABL TKI-resistant CML cell lines [15]. Therefore, the PI3K pathway is a suitable target for novel anticancer therapies, and the combination ABL TKI and PI3K inhibitor therapy may prove to be an effective therapeutic strategy for eliminating CML stem cells via BCR-ABL-dependent or -independent mechanisms. The PI3Kδ inhibitor, idelalisib was developed as the first of a new generation of oral agents for chronic lymphocyte leukemia. The available clinical data suggests that idelalisib has the potential to address the unmet need in elderly patients with CLL and may lead to remission [16]. Copanlisib, also known as BAY80-6946, is a potent, selective, and reversible inhibitor of PI3Kα and -δ [17]. Copanlisib has demonstrated broad antitumor activity, including anti-chronic lymphocytic leukemia (CLL) activity [18], and a Phase 2 study determining its efficacy against hematological malignancies is currently ongoing.

In this study, we investigated the effects of the specific PI3Kα and -δ inhibitor, copanlisib, against Ph-positive leukemia cells. We also investigated whether co-treatment with ABL TKIs and copanlisib could increase the cytotoxicity against Ph-positive leukemia cells, including ABL TKI-resistant samples.

## RESULTS

### Activity of the PI3K inhibitor copanlisib in a Ph-positive leukemia cell line

**Figure 1:**
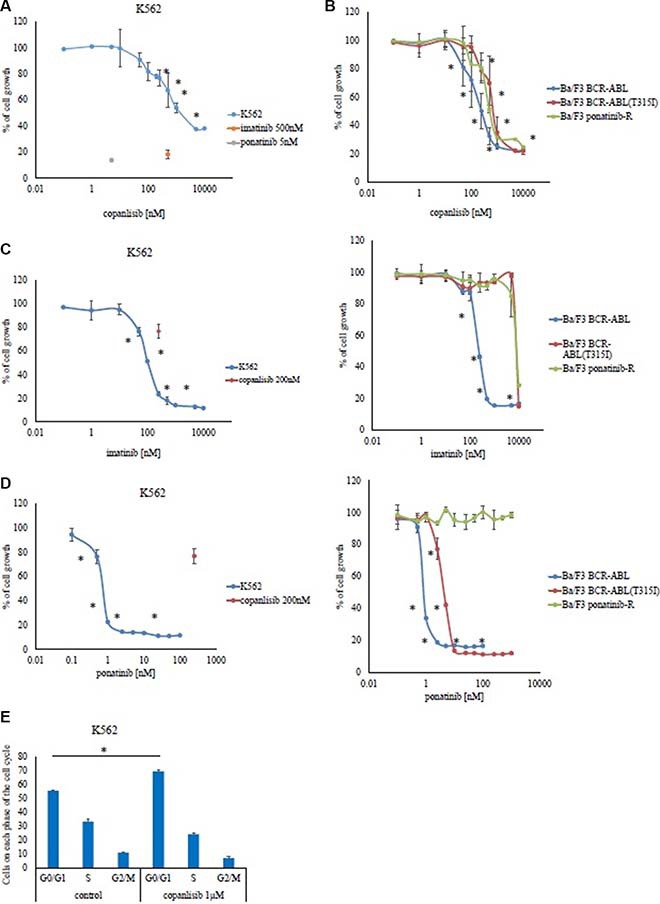
Effects of copanlisib and ABL TKI on BCR-ABL-positive cells K562 (**A**), as well as Ba/F3 BCR-ABL cells, Ba/F3 BCR-ABL (T315I) mutant cells, and Ba/F3 ponatinib-R cells (**B**) were treated with the indicated concentrations of copanlisib for 72 h, after which their relative growth rates was determined. *P < 0.05 compared with the control. K562, Ba/F3 BCR-ABL, Ba/F3 BCR-ABL (T315I) mutant cells, and Ba/F3 ponatinib-R cells were treated with the indicated concentrations of imatinib (**C**) or ponatinib (**D**) for 72 h, and their relative growth rates were determined. *P < 0.05 compared with the control. (**E**) A cell cycle analysis was performed as described in the Materials and Methods. The results (A–E) shown are representative of three independent experiments.

Copanlisib is a potent inhibitor of PI3Kα and -δ. First, we examined the activity of copanlisib in Ph-positive leukemia cells and found that it inhibited the growth of K562 (33% reduction at 500 nM compared with the control) and Ba/F3 BCR-ABL cells (65% reduction at 500 nM) (Figure 1A and 1B), in a dose dependent manner. Copanlisib also inhibited the growth of Ba/F3 BCR-ABL (T315I) mutant cells (30% reduction at 500 nM) (Figure 1B). Because patients who are resistant to ABL TKIs have poor prognosis, we investigated whether copanlisib inhibited the growth of ponatinib-resistant Ba/F3 cells (Ba/F3 ponatinib-R: BCR-ABL triple mutation: Y253H, E255K, and T315I). We found that copanlisib inhibited the proliferation of ABL TKI-resistant cells in a dose-dependent manner (50% reduction at 500 nM), as observed with the parental Ba/F3 BCR-ABL cells (Figure 1B). We also analyzed the cytotoxicity of ABL TKIs against these cell lines. Imatinib inhibited the growth of K562 (80% reduction at 500 nM) and Ba/F3 BCR-ABL cells (80% reduction at 500 nM), but not that of Ba/F3 BCR-ABL (T315I) mutant cells and Ba/F3 ponatinib-R cells (Figure 1C). In contrast, ponatinib inhibited the growth of K562 (78% reduction at 1 nM), Ba/F3 BCR-ABL (66% reduction at 1 nM), and Ba/F3 BCR-ABL (T315I) mutant cells (87% reduction at 10 nM) (Figure 1D). Cell survival differed at 24 h and 72 h post-treatment; thus, the efficacy of ponatinib was time-dependent. We also found that Ba/F3 ponatinib-R cells were resistant to ponatinib, at concentrations of up to 1 μM (Figure 1D). Flow cytometric analysis revealed that the percentage of G0/G1 was increased at 1 μM of copanlisib treatment (69%) in comparison to the control cells (55%) (Figure 1E, *p* < 0.05).

### The PI3K inhibitor copanlisib enhances ABL TKI activity in BCR-ABL-positive leukemia cells

**Figure 2:**
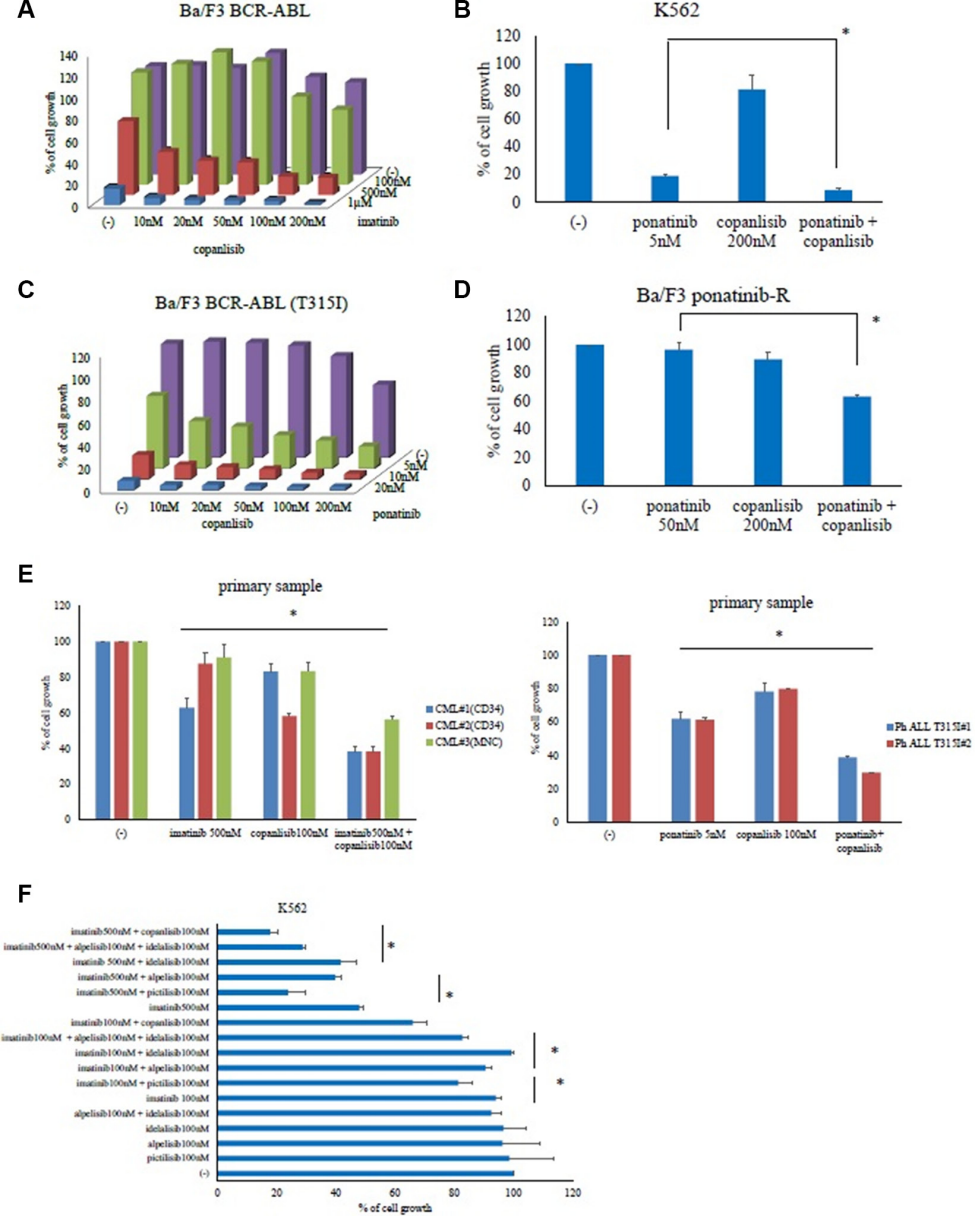
Co-treatment with copanlisib and ABL tyrosine kinase inhibitors decreased the proliferation of BCR-ABL-positive leukemia cells Ba/F3 BCR-ABL, K562, Ba/F3 BCR-ABL (T315I) mutant, and Ba/F3 ponatinib-R cells were treated with the indicated concentrations of copanlisib, imatinib (**A**), both, or ponatinib (**B**–**D**) for 72 h. The relative cell growth rates were determined. *P < 0.05 compared with ponatinib treatment. (**E**) CD34-positive CML cells, Ph-positive ALL T315I cells or CML mononuclear cells were treated with copanlisib, imatinib, both copanlisib and imatinib, or ponatinib for 72 h. The relative cell growth rates were determined. *P < 0.05, compared with the control cells. (**F**) K562 cells were treated with (i) imatinib and/or pictilisib, (ii) alpelisib, idelalisib, and imatinib, or (iii) with alpelisib and idelalisib for 72 h, after which the relative cell growth rates were determined. The data shown represent three independent sets of experiments. *P < 0.05, compared with alpelisib or idelalisib or pictilisib treatment alone. These experiments were performed in triplicate.

Copanlisib was tested in combination with imatinib against Ba/F3 BCR-ABL or K562 cells, revealing that the combination synergistically inhibited cell growth more than with either ABL TKI did alone (Figure 2A and Supplemental Figure S1A). Similar results were also obtained with the other ABL TKI, ponatinib (Figure 2B). Next, the combination of ponatinib and copanlisib treatment experiments was performed in Ba/F3 BCR-ABL (T315I) mutant cells. The ponatinib and copanlisib concentrations tested were 5–20 nM and 10–200 nM, respectively. Given that the plasma concentration of copanlisib was found to be up to 800 nM in a clinical trial [19], these conditions reflected clinically relevant concentrations. We found that the inhibition rate of ponatinib was 5 nM: 37% and copanlisib 50 nM: 2%. In contrast, 5 nM ponatinib plus 50 nM copanlisib inhibited 71% of the cell growth. This suggests that the combination treatment with ponatinib with copanlisib exhibited a synergistically enhanced cytotoxic effect in Ba/F3 BCR-ABL (T315I) mutant cells (Figure 2C). Subsequently, we found that the combination treatment with copanlisib and an ABL TKI (ponatinib) in ponatinib-resistant cells significantly inhibited cell proliferation (Figure 2D). Because copanlisib and ABL TKIs are promising therapeutic agents in Ph-positive leukemia cells (including those with the T315I mutation), we evaluated the efficacy of copanlisib in primary cells. Compared with the effects of monotherapy, co-treatment with copanlisib and imatinib or ponatinib significantly enhanced cytotoxicity in the Ph-positive primary samples (Figure 2E). Moreover, the combination treatment with both agents was effective in CD34-positive CML samples. We then examined whether the combined effects of ABL TKIs and copanlisib could be reproduced with other PI3K inhibitors (pictilisib, alpelisib, and idelalisib). We found that the combination treatment with imatinib and the pan-PI3K inhibitor, pictilisib inhibited cell growth, in contrast to the effects of each drug alone (Figure 2F). However, the efficacy of the specific PI3Kα inhibitor, alpelisib, or the PI3Kδ inhibitor, idelalisib, was lower than that of pictilisib. In contrast, co-treatment with imatinib and alpelisib plus idelalisib increased the inhibition of cell growth, suggesting that the dual inhibition of PI3Kα and -δ enhances ABL TKI activity.

### Efficacy of copanlisib and ABL TKIs in BCR-ABL-positive leukemia cells

**Figure 3:**
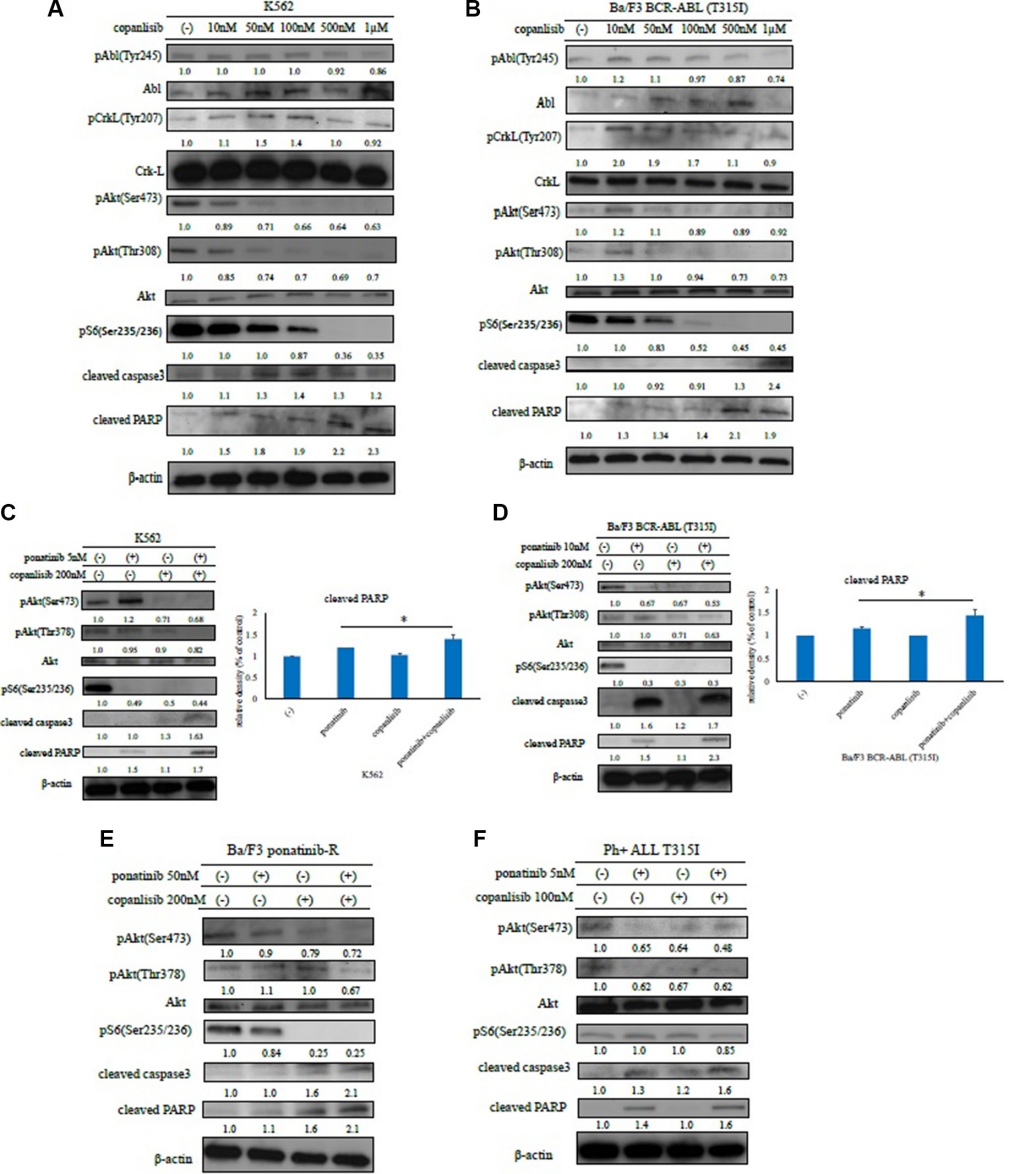
Effects of copanlisib and ABL TKIs on BCR-ABL-positive leukemia cells K562 (**A**) and Ba/F3 BCR-ABL (T315I) mutant cells (**B**) were treated with copanlisib at the indicated concentrations for 24 h. The total extracts were examined by an immunoblot analysis with Abs against phospho ABL (Tyr 245), phospho-Crk-L (Tyr207), phospho-Akt (Ser473), phospho-S6 ribosomal protein (Ser235/236), cleaved PARP, cleaved caspase 3, ABL, Crk-L, and β-actin. K562 cells (**C**), Ba/F3 BCR-ABL (T315I) mutant cells (**D**), Ba/F3 ponatinib-R cells (**E**), and primary cells (**F**) were treated with copanlisib and/or ponatinib at the indicated concentrations for 24 h. Cleaved PARP was quantified using ImageJ. *P < 0.05 compared with imatinib or ponatinib treatment. These experiments were performed in triplicate.

We next investigated the effects of copanlisib on intracellular signaling. A high concentration of copanlisib inhibited the phosphorylation of BCR-ABL, Crk-L, and Akt, and induced PARP activation in K562 and Ba/F3 BCR-ABL (T315I) mutant cells (Figure 3A and 3B). We also found that the co-treatment with ABL TKI and copanlisib reduced the phosphorylation of Akt and the ribosomal S6 protein, while increasing caspase 3 and PARP activity in K562 cells, Ba/F3 BCR-ABL (T315I) mutant cells, and Ba/F3 ponatinib-R cells (Figure 3C–3E). These results indicate that copanlisib and ABL TKI combination treatment are effective against ABL TKI-resistant cells. We next examined the intracellular signaling mechanisms in the primary samples. We found that the phosphorylation of Crk-L and S6 ribosomal protein decreased following treatment with copanlisib and ponatinib (Figure 3F). These findings indicate that the combination of copanlisib and ABL TKI was effective against Ph-positive primary samples.

### HS-5 feeder cells inhibit ABL TKI activity

**Figure 4:**
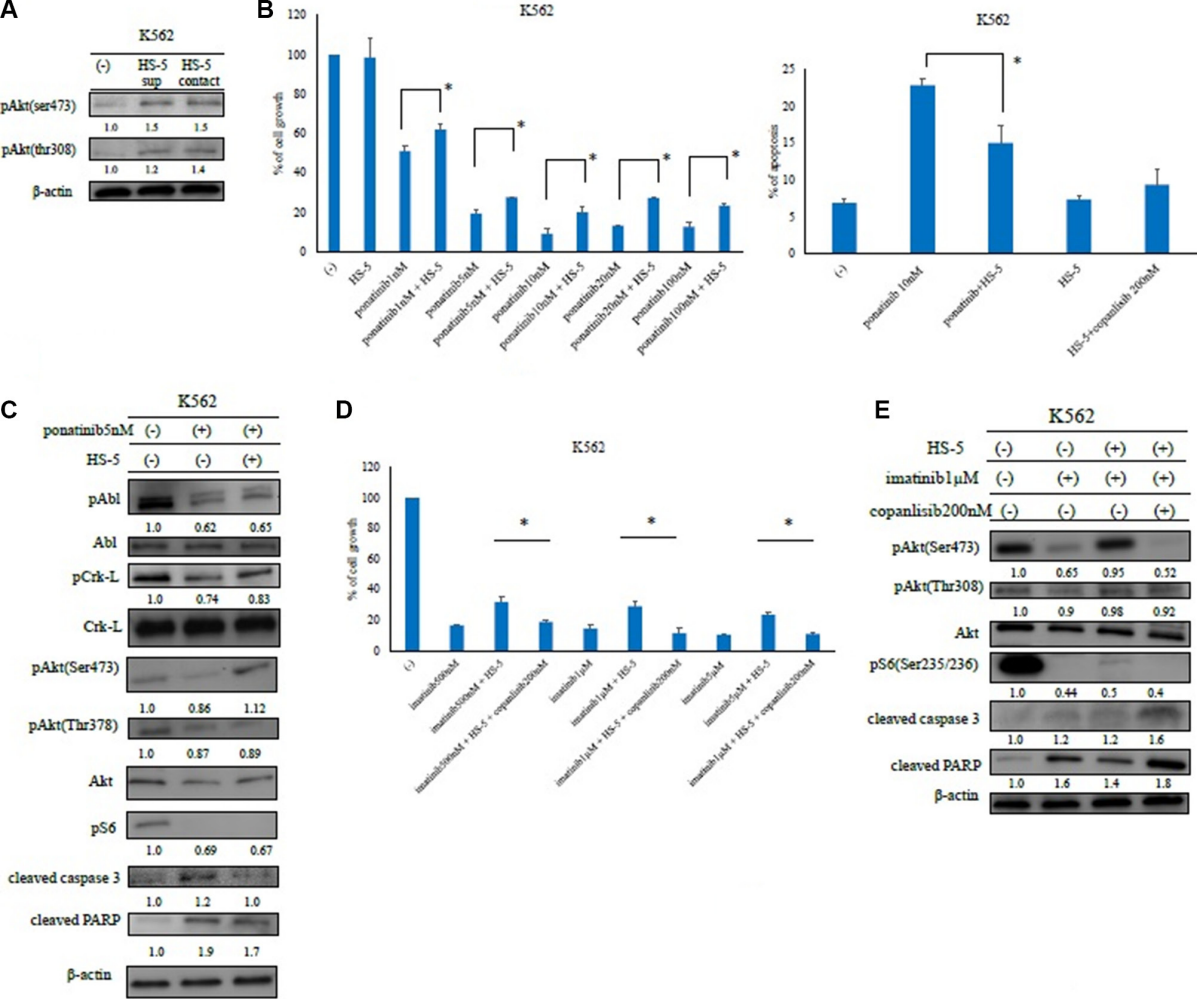
Analysis of ABL tyrosine kinase inhibitor activity in the presence of HS-5 feeder cells (**A**) K562 cells were treated with or without HS-5 cell culture supernatant and co-cultured with HS-5 cells for 24 h. Phosphorylation of Akt (Ser473 and Thr308) was examined by an immunoblot analysis. Actin was detected as a loading control. (**B**) K562 cells were co-cultured with or without HS-5 cells and treated with the indicated concentrations of ponatinib for 48 or 72 h, and the relative cell growth rates and percent of apoptotic cells were determined. *P < 0.05 compared with the control cells. The results shown represent three independent experiments. (**C**) K562 cells were co-cultured with or without HS-5 feeder cells and treated with ponatinib at the indicated concentration for 24 h. Total extracts were examined by an immunoblot analysis with Abs against phospho ABL (Tyr245), phospho-Crk-L (Tyr207), phospho-S6 ribosomal protein (Ser235/236), cleaved caspase 3, cleaved poly-ADP-ribose polymerase, ABL, Crk-L, and β-actin. (**D**, **E**) K562 cells were treated with copanlisib and imatinib in the presence of HS-5 feeder cells for 72 h, the relative cell growth rates were determined, and lysates were examined by an immunoblot analysis with Abs against phospho-Akt (Ser473, Thr308), phospho-S6 ribosomal protein (Ser235/236), cleaved PARP, cleaved caspase 3, and β-actin. These experiments were performed in triplicate.

In the local microenvironment, leukemia cells are surrounded by various types of stromal cells. These feeder cells can support leukemia cell growth. Therefore, we next examined the expression of the downstream effectors in K562 cells in the presence of the HS-5 feeder cell line. By conducting an immunoblot analysis, we found that Akt phosphorylation (Ser473 and Thr308) was reduced by ABL TKI treatment. In contrast, Akt phosphorylation increased in the presence of the HS-5 feeder cells (Figure 4A). Next, we evaluated the effects of ABL TKIs in the presence of the HS-5 feeder cells. K562 cells were cultured with or without ABL TKIs for 72 h. We found that the inhibition of K562 cell growth and apoptosis was significantly reduced in the presence of HS-5 feeder cells (Figure 4B, Supplemental Figure S1B). We also examined intracellular signaling and found that the phosphorylation of BCR-ABL, Crk-L, and S6 ribosomal protein (an Akt substrate), decreased, while caspase 3 and PARP activity increased following ABL TKI exposure (Figure 4C). In contrast, BCR-ABL and Crk-L phosphorylation increased, while caspase 3 and PARP activity decreased in the presence of HS-5 feeder cells. We then examined the activity of copanlisib in the presence of HS-5 cells. Although the activity of copanlisib decreased in the presence of HS-5 cells, K562 proliferation was significantly reduced by copanlisib, even in the presence of HS-5 cells (Figure 4D). We also found that the phosphorylation of Akt and the ribosomal S6 protein decreased, while caspase 3 and PARP activity increased following copanlisib treatment (Figure 4E).

### *In vivo* analysis of copanlisib and ponatinib activity

**Figure 5:**
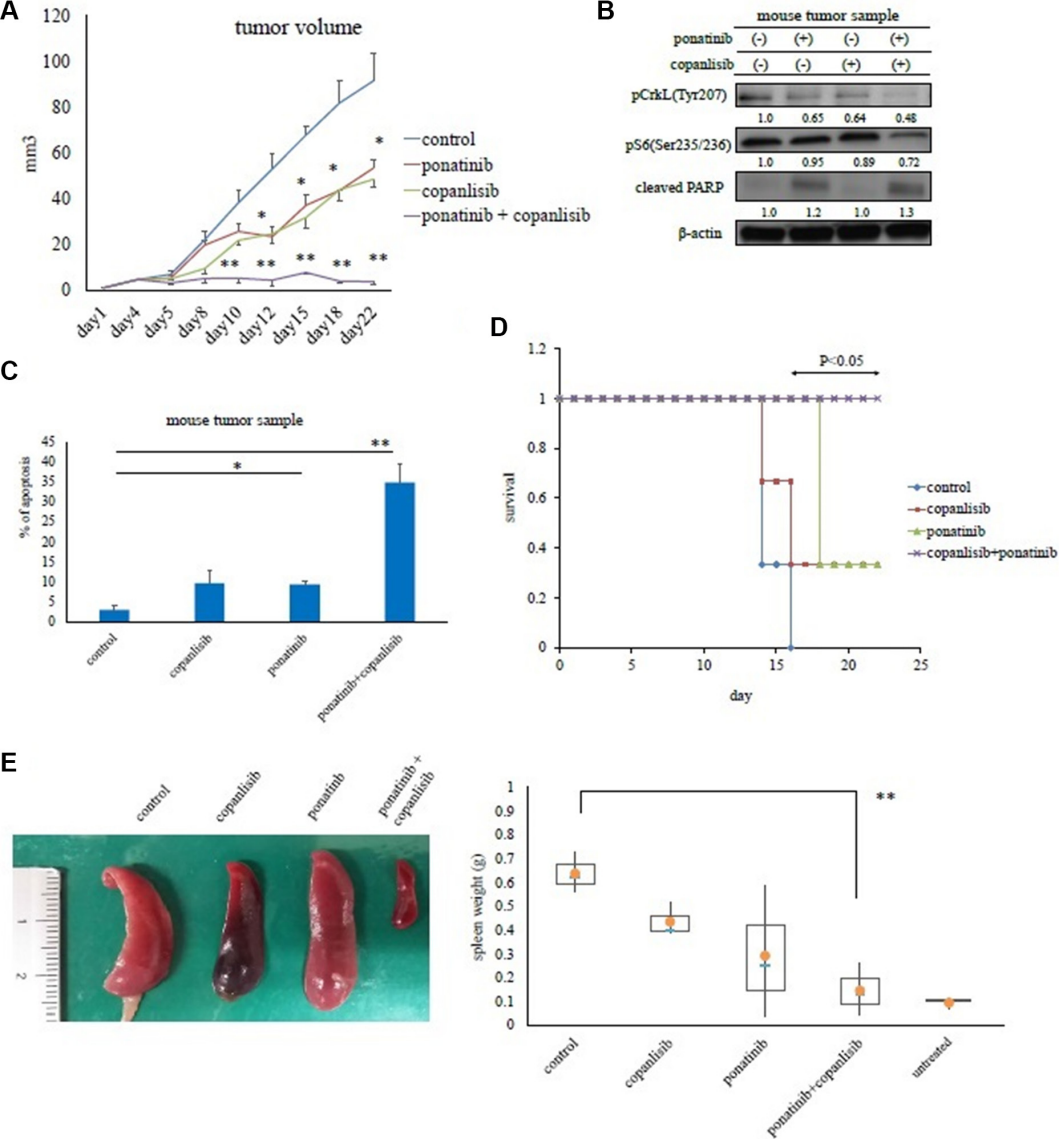
Effects of copanlisib and ponatinib on Ba/F3 T315I cell proliferation in a mouse model (**A**) *In vivo* studies were performed as described in the Materials and Methods. Tumor volumes were evaluated every three days. *P < 0.05 and **P < 0.01 compared with the control group (*n* = 4 mice/group). (**B**) Tumor samples from xenograft models treated with or without ponatinib and copanlisib were examined by an immunoblot analysis. (**C**) Tumor cells treated with or without copanlisib and ponatinib for 24 days were analyzed immunohistochemically via hematoxylin and eosin staining, or with an Ab against cleaved caspase 3, as described in the Materials and Methods. Cleaved caspase 3-positive cells in paraffin-embedded tissue sections of transplanted subcutaneous tumors were counted. *P < 0.05, **P < 0.01. (**D**) Kaplan–Meier survival curves for ponatinib- and/or copanlisib-treated Ba/F3 BCR-ABL (T315I) mutant cell intravenously injected mice (*n* = 3 mice/group). *P < 0.05 compared with control mice. (**E**) Total spleen volumes for intravenously injected and untreated control mice which did not receive transplanted Ba/F3 BCR-ABL (T315I) mutant cells. The image of the spleen is representative from each group of mice. All data are shown as the mean ± standard error of the mean from. **P < 0.01 compared with the control mice. The results shown in panels A–E are representative of at least two complete experiments.

The *in vivo* efficacy of copanlisib and the ABL TKI ponatinib were then evaluated using a mouse model. In this study, Ba/F3 BCR-ABL (T315I) mutant cells were injected subcutaneously or intravenously. The mice receiving the intravenous injection of the mutant cells developed a hematopoietic neoplasm and were used as an animal model of leukemia. Tumor size was evaluated every three days. Ponatinib administered orally (20 mg/kg; 5 days/week) or copanlisib administered intraperitoneally (6 mg/kg; 3 days/week) inhibited the growth of Ba/F3 BCR-ABL (T315I) mutant cells *in vivo* to a greater extent than the vehicle control (PBS) (*P* < 0.05). In addition, the combination of these drugs was significantly more effective (*P* < 0.01, Figure 5A). In tumor cells, the phosphorylation of Crk-L and the S6 ribosomal protein decreased, and PARP activity increased in ponatinib- and copanlisib-treated mice (Figure 5B). Combination treatment with copanlisib and ponatinib was also well tolerated in the treated mice. During immunohistochemical analysis, we found that tumors in mice treated with ponatinib and copanlisib exhibited an increase in apoptotic cells compared with the control mice (Figure 5C). We also found that co-treatment with copanlisib increased mouse survival and reduced the spleen size (Figure 5D and 5E).

## DISCUSSION

The PI3K signaling pathway plays a primary role in cell signaling and survival [14]. The PI3K p110δ subunit is selectively expressed in leukocytes and activates several downstream molecules. Feeder cells were found to maintain the residual leukemic cells, which were resistant to ABL TKIs. Furthermore, the HS-5 feeder cells provided adequate support for the Ph-positive samples (Figure 5A). LSCs are commonly observed in the bone marrow niche and are resistant to chemotherapy [20]; therefore, they must be eliminated to cure patients with hematological malignancies. In this study, we demonstrated that ABL TKI activity decreased, and that p110δ expression increased in the presence of HS-5 feeder cells. Our results indicate that PI3K, particularly p110δ, may be the target of Ph-positive leukemia cells when the feeder cells support the growth of the leukemia cells.

To model the effects of copanlisib in the BCR-ABL-mutant or ABL TKI-resistant cells, we studied several cell lines harboring the BCR-ABL T315I mutation, as well as ponatinib-resistant cells. Similar to our findings in K562 and Ba/F3 BCR-ABL cells, copanlisib was found to potently inhibit the proliferation of the ABL TKI-resistant cells. We also demonstrated that copanlisib enhanced ABL TKI activity. Moreover, copanlisib inhibited the PI3K/Akt pathway and mediated G0/G1 cell cycle arrest (Figure 1E). Because a single agent PI3K inhibitor could not block the BCR-ABL signaling pathway completely, the cell growth inhibition was partial, even at high concentrations of treatment. However, the combination of copanlisib and ponatinib tended to show a greater inhibition of cellular proliferation than that found after incubation with either drug alone. In particular, we demonstrated that copanlisib was highly effective against the T315I-mutant cells and enhanced the activity of ponatinib in the T315I-mutant mouse model (Figure 4A and 4B).

The PI3K signaling pathway controls the viability and proliferation of many cell types and is upregulated in human malignancies [14]. Several potential therapeutics targeting the PI3K signaling pathway have been evaluated against hematological malignancies, including in our previous studies [20–23]. Ding et al. reported that the inhibition of the PI3K/mTOR pathway overcame nilotinib-resistant Ph-positive cells via the downregulation of the mouse double minute 2 homolog [24]. Pellicano et al. reported that dasatinib and rapamycin or LY294002 decreased the phosphorylation of forkhead box O and induced apoptosis in CML cells [25]. Recently, idelalisib, an oral PI3K p110δ inhibitor, was investigated in a Phase 3 clinical trial in patients with indolent non-Hodgkin's lymphoma, CLL, and mantle cell lymphoma [26]. Moreover, the US Food and Drug Administration has approved idelalisib for use in treating CLL and indolent lymphoma. Furthermore, the combination of idelalisib and rituximab significantly improved the overall survival of patients with CLL [27]. Thus, the combination of these PI3K inhibitors demonstrates a tendency to improve treatment outcomes more than when individual drugs are administered alone.

Imatinib with pictilisib and alperlisib plus idelalisib appear to be more cytotoxic than treatment with isoform-specific inhibitors. A therapeutic advantage from targeting specific PI3K isoforms could depend on the balance between the efficacy in purging cancer and the deleterious side effects. Isoform-specific PI3K inhibitors may enable the reduction of side effects compared with pan-PI3K inhibitors and allow for higher tolerated doses in the clinic. However, the specific PI3Kα inhibitor, alpelisib or the PI3Kδ inhibitor, idelalisib could not enhance the ABL TKI activity. Importantly, our results indicate that the dual PI3Kα and–δ inhibitor, copanlisib may improve the outcome of patients with Ph-positive leukemia, including those with a T315I mutation. Because patients who exhibit ABL TKI resistance have a poor prognosis, we have already reported our findings in these patients, particularly those resistant to ponatinib [11]. There is new therapeutic approach against patients with ALL and CLL. For example, blinatumomab (MT103/MEDI-538), a first-in-class bispecific T engager (BiTE) antibody against CD19/CD3 for patients with relapsed/refractory ALL was developed [28]. ABT-199 (venetoclax, RG7601, GDC-0199) has also been granted breakthrough designation by FDA for relapsed or refractory CLL with a 17p deletion [29]. In this study, we demonstrated that copanlisib overcame the chemoprotective effect of feeder cells. Therefore, the inhibition of the PI3K pathway, particularly the activities of PI3Kα and -δ, may mediate beneficial therapeutic effects in hematological malignancies, including ABL TKI-resistant cells.

In conclusion, the results of our study indicate that the PI3K inhibitor copanlisib potentiates the activity of ABL TKIs in Ph-positive leukemia cells, including primary cells. Combination therapy with these two drug classes may improve the clinical outcome in patients with Ph-positive leukemia in the near future.

## MATERIALS AND METHODS

### Reagents

Copanlisib and ponatinib were purchased from MedKoo Biosciences (Chapel Hill, NC, USA). Imatinib and nilotinib were provided by Novartis Pharma AG (Basel, Switzerland). Stock solutions of nilotinib and ponatinib were prepared in dimethyl sulfoxide. Imatinib was dissolved in distilled water, aliquoted, and stored at −20°C. Copanlisib was dissolved in hydrochloric acid and diluted with distilled water. All other reagents were obtained from Sigma-Aldrich (St. Louis, MO, USA).

### Cell lines, patient samples, and CD34-positive samples

A Ph-positive leukemia cell line (K562) was obtained from the American Type Culture Collection (Manassas, VA, USA). ABL TKI-resistant cells, including Ba/F3 ponatinib-R (triple mutation: Y253H, E255K, and T315I), T315I mutant Ba/F3 BCR-ABL, and parental Ba/F3 BCR-ABL (wild type) were established [11, 30] previously. All cell lines were cultured in RPMI 1640 medium containing 10% fetal bovine serum and maintained at 37°C in a humidified atmosphere containing 5% CO_2_. Fresh peripheral blood samples (CML and Ph-positive leukemia cells) were collected from patients. Mononuclear cells were separated from the blood using LymphoSepare (Immuno-Biological Laboratories). These cells were either used immediately or cryopreserved in liquid nitrogen until use. In some experiments, CD34-positive cells were isolated using a CD34 MicroBead Kit (Miltenyi Biotec Inc., Auburn, CA, USA) according to the manufacturer's protocol. The study protocol was approved by the Institutional Review Board of Tokyo Medical University (No. 1974), and written informed consent was obtained from all patients in accordance with the Declaration of Helsinki.

### Cell viability assays

Cells were treated with copanlisib alone or in combination with ABL TKIs (imatinib, nilotinib, or ponatinib) and then treated with trypan blue (exclusion test) or stained using the Cell-Counting Kit (Dojindo, Kumamoto, Japan). Subsequently, photometrical measurements were taken at an absorbance of 450 nm to determine cell viability. All experiments were performed in triplicate.

### Immunoblotting

Immunoblot analysis was performed according to previously described methods [31]. Specific primary antibodies (Abs) against phospho-ABL (Tyr245), phospho-Crk-L (Tyr207), phospho-Akt (Ser473 and Thr308), phospho-S6 ribosomal protein (Ser235/236), p110α, cleaved caspase 3, and poly-ADP-ribose polymerase (PARP) were purchased from Cell Signaling Technology (Danvers, MA). The Crk-L Ab was purchased from EMD Millipore (Billerica, MA), while Abs against ABL and p110δ were from Santa Cruz Biotechnology (Santa Cruz, CA, USA). Three independent experiments were performed in each case. Determination of the concentration of protein in gel bands were performed using ImageJ.

### *In vivo* assays

Six-week-old female mice (BALB/c-nu) were subjected to subcutaneous or intravenous injection with 1 × 10^7^ Ba/F3 T315I cells. The mice were then treated with 20 mg/kg ponatinib five days a week (orally), 6 mg/kg copanlisib three times a week (intraperitoneally), or a combination of both agents in allograft models. The control mice were treated with phosphate-buffered saline (PBS) intraperitoneally. At various designated time points, the tumor size and mouse survival were recorded. The average tumor weight and spleen size per mouse were calculated and used to determine the group mean tumor volume or weight ± standard error of the mean (*n* = 4 or 3 mice) for each group. The total spleen volumes for intravenously injected and untreated control mice which did not receive transplanted Ba/F3 BCR-ABL (T315I) mutant cells was also investigated. Tumor cells were collected at the indicated times and fixed in paraformaldehyde. Paraffin-embedded tissues were stained with hematoxylin and eosin, or an Ab against cleaved caspase 3, and the number of apoptotic cells was calculated.

### Cell cycle analysis

Cell cycle analysis was performed using the BD Cycletest™ Plus DNA kit (BD Biosciences, Franklin Lakes, NJ, USA) in the manufacture's protocol. After 48 h of treatment, the effect of copanlisib on the cell cycle distribution in K562 cells was analyzed by flow cytometry.

### Statistical analysis

A Student's *t-test* and two-way analysis of variance were used to determine whether the effects of drug treatment were statistically significant compared with those in the control group. A probability (*P*) value < 0.05 was considered to be statistically significant.

## SUPPLEMENTARY MATERIAL FIGURE



## References

[1] Rowley JD (1973). Letter: A new consistent chromosomal abnormality in chronic myelogenous leukemia identified by quinacrine fluorescence and Giemsa staining. Nature.

[2] Kantarjian HM, Talpaz M, Giles F, O'Brien S, Cortes J (2006). New insights into the pathophysiology of chronic myeloid leukemia and imatinib resistance. Ann Intern Med.

[3] Kantarjian H, Sawyers C, Hochhaus A, Guilhot F, Schiffer C, Gambacorti-Passerini C, Niederwieser D, Resta D, Capdeville R, Zoellner U, Talpaz M, Druker B, Goldman J (2002). Hematologic and cytogenetic responses to imatinib mesylate in chronic myelogenous leukemia. N Engl J Med.

[4] Hochhaus A, O'Brien SG, Guilhot F, Druker BJ, Branford S, Foroni L, Goldman JM, Müller MC, Radich JP, Rudoltz M, Mone M, Gathmann I, Hughes TP (2009). Six-year follow-up of patients receiving imatinib for the first-line treatment of chronic myeloid leukemia. Leukemia.

[5] Kantarjian HM, Giles F, Gattermann N, Bhalla K, Alimena G, Palandri F, Ossenkoppele GJ, Nicolini FE, O'Brien SG, Litzow M, Bhatia R, Cervantes F, Haque A (2007). Nilotinib (formerly AMN107), a highly selective BCR-ABL tyrosine kinase inhibitor, is effective in patients with Philadelphia chromosome-positive chronic myelogenous leukemia in chronic phase following imatinib resistance and intolerance. Blood.

[6] Shah NP, Kantarjian HM, Kim DW, Réa D, Dorlhiac-Llacer PE, Milone JH, Vela-Ojeda J, Silver RT, Khoury HJ, Charbonnier A, Khoroshko N, Paquette RL, Deininger M (2008). Intermittent target inhibition with dasatinib 100 mg once daily preserves efficacy and improves tolerability in imatinib-resistant and -intolerant chronic-phase chronic myeloid leukemia. J Clin Oncol.

[7] Saglio G, Kim DW, Issaragrisil S, le Coutre P, Etienne G, Lobo C, Pasquini R, Clark RE, Hochhaus A, Hughes TP, Gallagher N, Hoenekopp A, Dong M (2010). Nilotinib versus imatinib for newly diagnosed chronic myeloid leukemia. N Engl J Med.

[8] Kantarjian H, Shah NP, Hochhaus A, Cortes J, Shah S, Ayala M, Moiraghi B, Shen Z, Mayer J, Pasquini R, Nakamae H, Huguet F, Boqué C (2010). Dasatinib versus imatinib in newly diagnosed chronic-phase chronic myeloid leukemia. N Engl J Med.

[9] Soverini S, De Benedittis C, Papayannidis C, Paolini S, Venturi C, Iacobucci I, Luppi M, Bresciani P, Salvucci M, Russo D, Sica S, Orlandi E, Intermesoli T (2014). Drug resistance and BCR-ABL kinase domain mutations in Philadelphia chromosome-positive acute lymphoblastic leukemia from the imatinib to the second-generation tyrosine kinase inhibitor era: The main changes are in the type of mutations, but not in the frequency of mutation involvement. Cancer.

[10] Cortes JE, Kantarjian H, Shah NP, Bixby D, Mauro MJ, Flinn I, O'Hare T, Hu S, Narasimhan NI, Rivera VM, Clackson T, Turner CD, Haluska FG (2012). Ponatinib in refractory Philadelphia chromosome-positive leukemias. N Engl J Med.

[11] Okabe S, Tauchi T, Tanaka Y, Katagiri S, Kitahara T, Ohyashiki K (2013). Activity of omacetaxine mepesuccinate against ponatinib-resistant BCR-ABL-positive cells. Blood.

[12] Stein AM, Bottino D, Modur V, Branford S, Kaeda J, Goldman JM, Hughes TP, Radich JP, Hochhaus A (2011). BCR-ABL transcript dynamics support the hypothesis that leukemic stem cells are reduced during imatinib treatment. Clin Cancer Res.

[13] Vanhaesebroeck B, Guillermet-Guibert J, Graupera M, Bilanges B (2010). The emerging mechanisms of isoform-specific PI3K signaling. Nat Rev Mol Cell Biol.

[14] Thorpe LM, Yuzugullu H, Zhao JJ (2015). PI3K in cancer: divergent roles of isoforms, modes of activation and therapeutic targeting. Nat Rev Cancer.

[15] Quentmeier H, Eberth S, Romani J, Zaborski M, Drexler HG (2011). BCR-ABL1-independent PI3Kinase activation causing imatinib-resistance. J Hematol Oncol.

[16] Rai KR (2015). Therapeutic potential of new B cell-targeted agents in the treatment of elderly and unfit patients with chronic lymphocytic leukemia. J Hematol Oncol.

[17] Liu N, Rowley BR, Bull CO, Schneider C, Haegebarth A, Schatz CA, Fracasso PR, Wilkie DP, Hentemann M, Wilhelm SM, Scott WJ, Mumberg D, Ziegelbauer K (2013). BAY 80–6946 is a highly selective intravenous PI3K inhibitor with potent p110α and p110δ activities in tumor cell lines and xenograft models. Mol Cancer Ther.

[18] Göckeritz E, Kerwien S, Baumann M, Wigger M, Vondey V, Neumann L, Landwehr T, Wendtner CM, Klein C, Liu N, Hallek M, Frenzel LP, Krause G (2015). Efficacy of phosphatidylinositol-3 kinase inhibitors with diverse isoform selectivity profiles for inhibiting the survival of chronic lymphocytic leukemia cells. Int J Cancer.

[19] Doi T, Fuse N, Yoshino T, Kojima T, Bando H, Miyamoto H, Kaneko M, Osada M, Ohtsu A (2013). Abstract 29: Phase I study of intravenous PI3K inhibitor BAY 80–6946 in Japanese subjects. Cancer Res.

[20] Tabe Y, Konopleva M (2014). Advances in understanding the leukemia microenvironment. Br J Haematol.

[21] Burchert A, Wang Y, Cai D, von Bubnoff N, Paschka P, Müller-Brüsselbach S, Ottmann OG, Duyster J, Hochhaus A, Neubauer A (2005). Compensatory PI3-kinase/Akt/mTor activation regulates imatinib resistance development. Leukemia.

[22] Airiau K, Mahon FX, Josselin M, Jeanneteau M, Belloc F (2013). PI3K/mTOR pathway inhibitors sensitize chronic myeloid leukemia stem cells to nilotinib and restore the response of progenitors to nilotinib in the presence of stem cell factor. Cell Death Dis.

[23] Okabe S, Tauchi T, Tanaka Y, Kitahara T, Kimura S, Maekawa T, Ohyashiki K (2014). Efficacy of the dual PI3K and mTOR inhibitor NVP-BEZ235 in combination with nilotinib against BCR-ABL-positive leukemia cells involves the ABL kinase domain mutation. Cancer Biol Ther.

[24] Ding J, Romani J, Zaborski M, MacLeod RA, Nagel S, Drexler HG, Quentmeier H (2013). Inhibition of PI3K/mTOR overcomes nilotinib resistance in BCR-ABL1 positive leukemia cells through translational down-regulation of MDM. PLoS One.

[25] Pellicano F, Scott MT, Helgason GV, Hopcroft LE, Allan EK, Aspinall-O'Dea M, Copland M, Pierce A, Huntly BJ, Whetton AD, Holyoake TL (2014). The antiproliferative activity of kinase inhibitors in chronic myeloid leukemia cells is mediated by FOXO transcription factors. Stem Cells.

[26] Gopal AK, Kahl BS, Vos S, Wagner-Johnston ND, Schuster SJ, Jurczak WJ, Flinn IW, Flowers CR, Martin P, Viardot A, Blum KA, Goy AH, Davies AJ (2014). PI3Kδ inhibition by idelalisib in patients with relapsed indolent lymphoma. N Engl J Med.

[27] Furman RR, Sharman JP, Coutre SE, Cheson BD, Pagel JM, Hillmen P, Barrientos JC, Zelenetz AD, Kipps TJ, Flinn I, Ghia P, Eradat H, Ervin T (2014). Idelalisib and rituximab in relapsed chronic lymphocytic leukemia. N Engl J Med.

[28] Wu J, Fu J, Zhang M, Liu D (2015). Blinatumomab: a bispecific T cell engager (BiTE) antibody against CD19/CD3 for refractory acute lymphoid leukemia. J Hematol Oncol.

[29] Cang S, Iragavarapu C, Savooji J, Song Y, Liu D (2015). ABT-199 (venetoclax) and BCL-2 inhibitors in clinical development. J Hematol Oncol.

[30] Kimura S, Naito H, Segawa H, Kuroda J, Yuasa T, Sato K, Yokota A, Kamitsuji Y, Kawata E, Ashihara E, Nakaya Y, Naruoka H, Wakayama T (2005). NS-187, a potent and selective dual Bcr-Abl/Lyn tyrosine kinase inhibitor, is a novel agent for imatinib-resistant leukemia. Blood.

[31] Okabe S, Tauchi T, Kimura S, Maekawa T, Kitahara T, Tanaka Y, Ohyashiki K (2014). Combining the ABL1 kinase inhibitor ponatinib and the histone deacetylase inhibitor vorinostat: a potential treatment for BCR-ABL-positive leukemia. PLoS One.

