# Supplementary material for: Combination therapy with copanlisib and ABL tyrosine kinase inhibitors against Philadelphia chromosome-positive resistant cells
Source: Oncotarget. 2016 Jul 14;7(33):53116–26. doi: 10.18632/oncotarget.10605 (PMC5288172; doi:10.18632/oncotarget.10605)
Supplement: Supplementary file 1 [file oncotarget-07-53116-s001.pdf]

Combination therapy with copanlisib and ABL tyrosine kinase inhibitors against Philadelphia chromosome-positive resistant cells

Supplementary Materials

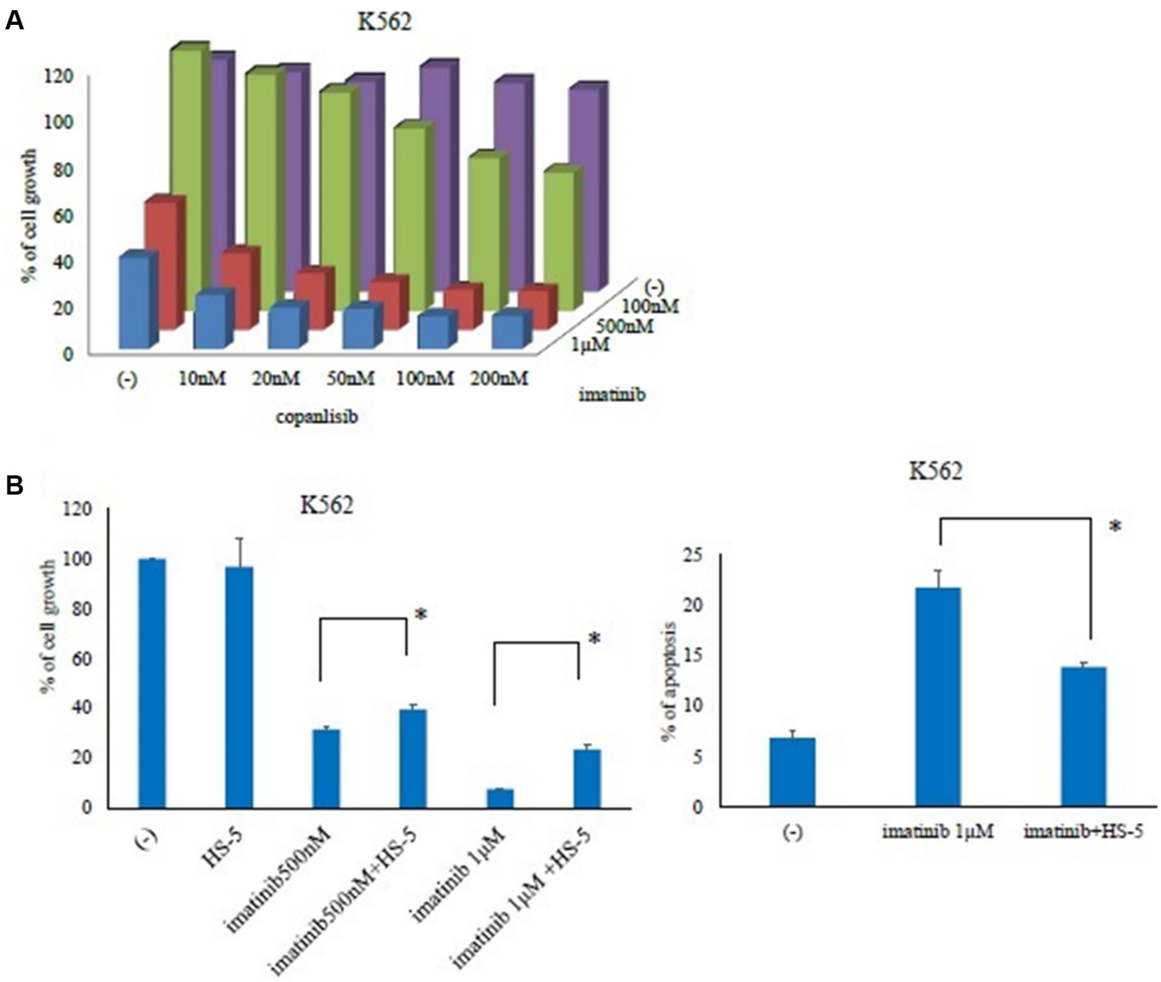

Supplementary Figure S1: Effect of copanlisib and imatinib on K562 and imatinib activity in the presence of HS-5 feeder cells. (**A**) K562 cells were treated with the indicated concentrations of copanlisib, imatinib, or both for 72 h. The relative cell growth rates were determined as described in the Materials and Methods section. The data shown represent three independent sets of experiments. (**B**) K562 cells were co-cultured with or without HS-5 and treated with indicated concentrations of imatinib for 72 h. Relative cell growth rates and the percentages of apoptotic cells were determined. *$P < 0.05$ compared with the data obtained following imatinib treatment. These experiments were performed in triplicate.
